# Citrullinated histone H3 identifies neutrophil extracellular trap formation and correlates with renal disease activity in ANCA-associated vasculitis

**DOI:** 10.1093/ckj/sfag110

**Published:** 2026-04-06

**Authors:** Natsumi Kamijo, Akiko Mii, Takashi Tani, Rei Nakazato, Arimi Ishikawa, Tetsuya Kashiwagi, Ryuji Ohashi, Masataka Kuwana, Akira Shimizu, Yukinao Sakai, Masato Iwabu

**Affiliations:** Department of Endocrinology, Metabolism and Nephrology, Nippon Medical School, Tokyo, Japan; Department of Endocrinology, Metabolism and Nephrology, Nippon Medical School, Tokyo, Japan; Department of Endocrinology, Metabolism and Nephrology, Nippon Medical School, Tokyo, Japan; Department of Endocrinology, Metabolism and Nephrology, Nippon Medical School, Tokyo, Japan; Department of Analytic Human Pathology, Nippon Medical School, Tokyo, Japan; Department of Endocrinology, Metabolism and Nephrology, Nippon Medical School, Tokyo, Japan; Department of Integrated Diagnostic Pathology, Nippon Medical School, Tokyo, Japan; Department of Allergy and Rheumatology, DNippon Medical School, Tokyo, Japan; Department of Analytic Human Pathology, Nippon Medical School, Tokyo, Japan; Department of Endocrinology, Metabolism and Nephrology, Nippon Medical School, Tokyo, Japan; Department of Endocrinology, Metabolism and Nephrology, Nippon Medical School, Tokyo, Japan

**Keywords:** ANCA-associated vasculitis, biomarker, citrullinated histone H3, neutrophil extracellular traps, renal biopsy

## Abstract

**Background:**

Antineutrophil cytoplasmic antibody-associated vasculitis (AAV) often manifests with necrotizing glomerulonephritis. Neutrophil extracellular traps (NETs) are implicated in its pathogenesis; citrullinated histone H3 (H3Cit) is a specific marker of NET formation. To date, no studies have assessed the relationship between H3Cit-positive cells and disease activity in patients with AAV. In this study, we assessed whether H3Cit-positive cells in renal tissue were associated with disease activity.

**Methods:**

We retrospectively evaluated 50 patients newly diagnosed with AAV by renal biopsy between January 2011 and August 2024. Paraffin-embedded specimens underwent immunohistochemical staining for H3Cit. Patients were classified as H3Cit-positive or H3Cit-negative groups based on the presence of H3Cit-positive cells in glomeruli and/or interstitium. Clinical parameters and histopathological features were compared. Additionally, correlations between H3Cit-positive cell counts and urinary biomarkers were evaluated.

**Results:**

H3Cit-positive cells were detected in 42 cases (84%) within the interstitium; of these 42 cases, they were also detected in 23 within the glomeruli. Compared with H3Cit-negative cases, the positive group had significantly higher urinary β_2_-microglobulin (β_2_-MG) and N-acetyl-β-D-glucosaminidase index values. Interstitial H3Cit-positive cell counts positively correlated with urinary β_2_-MG levels. The positive group showed more frequent crescent formation and peritubular capillaritis, and H3Cit-positive cells were present in all arteritic lesions.

**Conclusions:**

H3Cit-positive cells in renal tissue were associated with active glomerular and tubulointerstitial lesions in AAV cases and correlated with urinary markers of interstitial injury. H3Cit immunostaining may serve as a pathological marker of renal disease activity in AAV cases and support clinical assessment at the time of biopsy.

KEY LEARNING POINTS **What was known:**

- Neutrophil extracellular traps (NETs) are implicated in the pathogenesis of antineutrophil cytoplasmic antibody-associated vasculitis (AAV).
- We previously demonstrated that immunohistochemical staining for citrullinated histone H3 (H3Cit), a specific marker of NETs, can successfully visualize NET formation in paraffin-embedded renal biopsy sections, and that H3Cit-positive cells are highly specific for AAV.
- However, the clinical and pathological significance of H3Cit expression in relation to renal disease activity has not been fully clarified.

**This study adds:**

- The extent of H3Cit-positive cells in renal tissue correlates with histological indices of active glomerular and interstitial injury in AAV.
- H3Cit expression also correlates with urinary markers of tubular and interstitial injury.
- These findings indicate that H3Cit staining reflects ongoing NET formation and renal disease activity in AAV.

**Potential impact:**

- Immunohistochemical detection of H3Cit provides a feasible and reproducible approach to visualize NET formation *in situ*.
- H3Cit immunostaining may serve as a pathological marker of renal disease activity and support clinical assessment at the time of biopsy.
- Incorporating H3Cit evaluation into renal pathology studies could help recognize active vasculitis-related injury and contribute to a better understanding of NET-mediated renal inflammation.

## INTRODUCTION

Antineutrophil cytoplasmic antibody (ANCA)-associated vasculitis (AAV) is an autoimmune disease characterized by necrotizing inflammation of small-to-medium-sized blood vessels [1]. Based on clinicopathological features, AAV is classified into granulomatosis with polyangiitis (GPA), microscopic polyangiitis (MPA), eosinophilic GPA (EGPA), and renal-limited vasculitis [2]. AAV affects multiple organs, most commonly the lungs and kidneys, often with life-threatening manifestations such as pulmonary hemorrhage and rapidly progressive glomerulonephritis [3]. Neutrophils play a central role in AAV initiation and progression. They serve not only as the primary targets of ANCA but also as potent effectors of vascular endothelial injury [4]. The principal target antigens are proteinase 3 (PR3) and myeloperoxidase (MPO), which are normally localized in the cytoplasmic granules of neutrophils [5]. Upon priming by infectious or environmental stimuli, PR3 and MPO translocate to the cell surface, where ANCA binding triggers robust neutrophil activation. This leads to degranulation and the release of reactive oxygen species, proteolytic enzymes, and neutrophil extracellular traps (NETs), collectively mediating endothelial damage [4–7].

NETs, first described in 2004, were initially recognized for their role in trapping bacteria and exerting antimicrobial activity [8]. They are web-like structures composed of DNA fibers, histones, and antimicrobial proteins such as MPO and neutrophil elastase released from activated neutrophils. NET formation is often associated with a unique form of neutrophil cell death termed NETosis and contributes not only to infection control, but also to the development of autoimmune diseases, thrombosis, and other disorders [9, 10]. NETs have also been implicated in exacerbating organ injury, including cancer-associated thrombosis and kidney injury [11, 12]. Accumulating evidence links NETs directly to AAV pathogenesis. Several studies have demonstrated that neutrophils treated with serum from patients with AAV produce excessive NETs [13, 14]. In patients with AAV, NETs have been identified in sites of active vasculitis such as the kidneys, skin, and peripheral nerves [9, 15].

Histone citrullination is a crucial step in NET formation and represents a key molecular event driving chromatin decondensation during NETosis. Peptidylarginine deiminase 4 (PAD4) catalyzes the conversion of arginine residues in histone H3 to citrulline, resulting in histone hypercitrullination and facilitating the release of decondensed chromatin during NET formation. Thus, citrullinated histone H3 (H3Cit) reflects a key molecular event in the NETosis pathway and has been widely used as a surrogate marker of NET formation. H3Cit-positive cells are widely regarded as a marker of PAD4-dependent neutrophil activation and NET formation. [6, 10, 16]. However, extracellular trap formation is not strictly limited to neutrophils. Histone citrullination may also occur in other myeloid cells under inflammatory conditions, including macrophage-derived extracellular trap (MET) formation [17].

Recent studies have suggested that H3Cit may serve as a specific marker for NET formation in human tissue specimens and blood samples [16, 18–20]. Elevated circulating levels of H3Cit, MPO, and PAD4 have been reported in patients with AAV, reflecting enhanced NETosis activity in active vasculitis [21]. Experimental animal models further support these findings, demonstrating that circulating H3Cit levels correlate with disease activity; neutralization of H3Cit may have therapeutic potential [22]. In our previous study, we performed H3Cit immunostaining in various types of proliferative nephritis with neutrophil infiltration, including AAV, by immunostaining paraffin-embedded renal biopsy sections for H3Cit [23]. We quantitatively analyzed the frequency of H3Cit-positive cells among MPO-positive cells and found that H3Cit-positive cells were significantly enriched in AAV compared with other types of glomerulonephritis, including lupus nephritis. While glomerular H3Cit-positive cells were occasionally observed across disease groups, their relative frequency and spatial distribution significantly differed from those in AAV, supporting disease specificity of H3Cit-positive cells for AAV. However, whether these H3Cit-positive cells correlate with disease activity or histopathological severity in AAV remains unclear. Therefore, we aimed to clarify whether the presence of H3Cit-positive cells in renal tissue could serve as a useful marker for evaluating disease activity in patients with AAV.

## MATERIALS AND METHODS

### Participants

**Figure 1:**
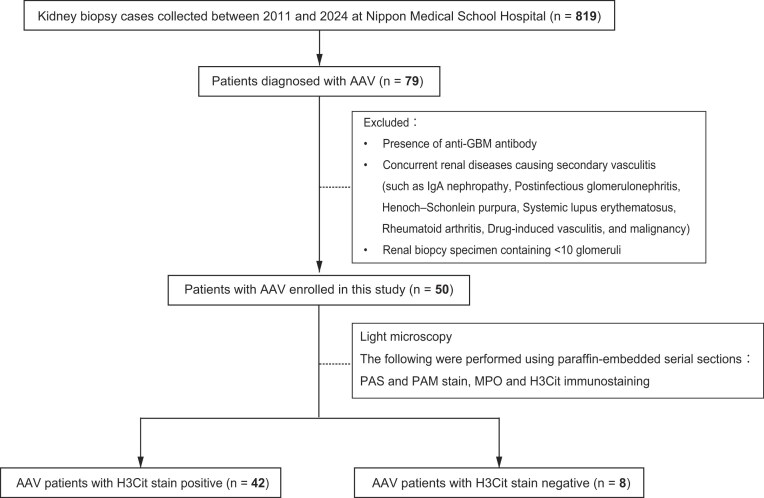
Flowchart of study participants.

In this retrospective observational study, we included patients newly diagnosed with AAV by renal biopsy at Nippon Medical School Hospital between January 2011 and August 2024. As illustrated in Fig. 1, cases were selected from all those who underwent renal biopsies at our institution between 2001 and 2024 (*n* = 819); from which, those meeting the diagnostic criteria for AAV (*n* = 79) were identified. After applying the following exclusion criteria, a total of 50 cases were finally included. The exclusion criteria were as follows: positivity for antiglomerular basement membrane antibodies; coexistence of other renal diseases that could cause secondary vasculitis (IgA nephropathy, IgA vasculitis known as Henoch–Schönlein purpura nephritis, postinfectious glomerulonephritis, systemic lupus erythematosus, rheumatoid arthritis under treatment, or drug-induced vasculitis); concurrent malignancy; and renal biopsy specimens containing fewer than 10 glomeruli. All patients were classified according to the 2012 revised International Chapel Hill Consensus Conference nomenclature into those with MPA (*n* = 48), GPA (*n* = 1), and EGPA (*n* = 1). The study protocol was approved by the Human Ethics Review Committee of Nippon Medical School (Approval number: M-2024-222). We obtained informed consent in the form of opt-out on the Nippon Medical School website.

### Clinical measurements

Demographic and clinical data, including age, sex, and presence of hypertension, were collected from medical records at the time of renal biopsy diagnosis. Laboratory data included serum creatinine (sCr; mg/dl), estimated glomerular filtration rate (eGFR; ml/min/1.73 m^2^), hemoglobin (Hb; mg/dl), serum albumin (Alb; mg/dl), C-reactive protein (CRP; mg/dl), MPO-ANCA (IU/ml), C3 (mg/dl), C4 (mg/dl), total hemolytic complement activity (CH50; U/ml), urinary β_2_-microglobulin (uβ_2_-MG; mg/dl), N-acetyl-β-D-glucosaminidase index (NAG index; U/g Cr), urinary protein (g/g Cr), and urinary red blood cell count [per selected high-power fields (HPFs)]. eGFR was calculated using the following formula validated for Japanese individuals:


\begin{eqnarray*}
{\mathrm{eGFR}}({\mathrm{ml}}/{\mathrm{min}}/1.73\,{{\mathrm{m}}}^2) &=& 194 \times {\mathrm{C}}{{\mathrm{r}}}^{ - 1.094}\\&&\times {\mathrm{ag}}{{\mathrm{e}}}^{ - 0.287}( \times 0.7399\,{\mathrm{in}}\,{\mathrm{females}})
\end{eqnarray*}

### Renal histopathology

For routine diagnostic evaluation by light microscopy, renal tissue samples were fixed in 20% buffered formaldehyde, embedded in paraffin, and stained with periodic acid–Schiff (PAS) and periodic acid–methenamine silver (PAM). Additionally, immunohistochemical staining (IHC) for H3Cit and MPO was performed on serial sections of formalin-fixed, paraffin-embedded (FFPE) renal biopsy specimens. For H3Cit, two rabbit antibodies, ab281584 (Abcam, Cambridge, UK) and #97272 (Cell Signaling Technology, Danvers, MA, USA), were initially evaluated in preliminary experiments. As no substantial differences in staining patterns were observed between the two antibodies (Supplemental Fig. 1), the Abcam antibody (ab281584) was used for subsequent analyses and for serial-section comparison with PAS, PAM, and MPO staining. MPO staining was performed using anti-MPO (A398; Dako, Glostrup, Denmark). For antigen retrieval, heat-induced antigen retrieval was performed by autoclave treatment using Histofine Antigen Retrieval Solution (pH 6.0; Nichirei Biosciences Inc., Tokyo, Japan) for MPO staining and Histofine Antigen Retrieval Solution (pH 9.0; Nichirei Biosciences Inc., Tokyo, Japan) for H3Cit staining. Detection was carried out using Simple Stain MAX-PO system (Nichirei Biosciences Inc., Tokyo, Japan). For evaluation of immunoglobulin and complement deposition, frozen tissue sections were processed for immunofluorescence (IF) using fluorescein isothiocyanate-conjugated antibodies against IgG, IgM, IgA, C3, C1q, and C4 (all from MBL, Nagoya, Japan).

Renal biopsy specimens were evaluated for the following histopathological parameters: glomerular necrosis (present/absent); crescent formation rate, defined as the percentage of cellular and fibrocellular crescents among all glomeruli; percentage of globally sclerotic glomeruli; interstitial fibrosis and tubular atrophy (IFTA; %); peritubular capillaritis (PTC-itis; present/absent); renal tubulitis (present/absent); arteritis (present/absent); and arteriosclerosis score (0 = none, 1 = mild, 2 = moderate, 3 = severe).

H3Cit-positive cells were counted in all glomeruli; the mean number per glomerulus was calculated. Ten randomly selected HPFs (×400) were analyzed to determine the mean number of H3Cit-positive cells per field for interstitial evaluation.

Complement immunostaining intensity was independently assessed by two renal pathologists blinded to the clinical data, using a four-tier scale: negative (−), weak (±), moderate (1+), and strong (2+). IF was considered positive when the score was ≥1+. The frequency of glomerular C3 positivity on routine diagnostic IF was subsequently compared between the H3Cit-positive and H3Cit-negative groups.

### Statistical analyses

All statistical analyses were performed using the Excel Statistics software (version 8.0; Esumi Co., Ltd, Tokyo, Japan). Data are presented as median and interquartile range. Differences in continuous variables between groups were evaluated using the nonparametric Mann–Whitney U test, whereas categorical variables were compared using Fisher’s exact test. The correlation between uβ_2_-MG levels and the number of H3Cit-positive cells in the interstitium was evaluated using Spearman’s rank correlation test. To account for multiple comparisons, *q*-values were calculated using the Benjamini–Hochberg false discovery rate method. The *q*‐value was calculated using the following formula: *q* = *P* × *N*/*i* (where *P* is the *P*‐value, *N* is the total number of hypotheses tested, and *i* is the rank of the *P*‐value). A *P*-value < .05 was considered statistically significant. For multiple comparisons, statistical significance was defined as *q* < 0.05.

## RESULTS

### Clinical characteristics

**Figure 2:**
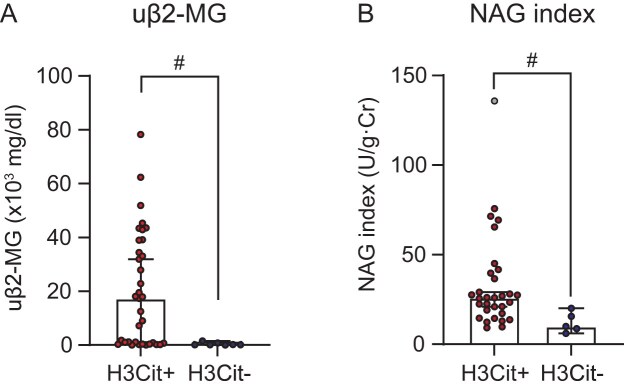
Comparison of urinary tubular injury markers between H3Cit-positive and H3Cit-negative groups. (A) uβ₂-MG levels were significantly higher in the H3Cit-positive group than in the H3Cit-negative group. (B) Similarly, the NAG index (U/g Cr) value, a marker of tubular injury, was significantly higher in the H3Cit-positive group than in the H3Cit-negative group. Data are presented as median ± interquartile range. #*P* < .01 by the Mann–Whitney U test. Gray dot indicates an outlier.

Immunohistochemistry for H3Cit was performed on paraffin-embedded sections of renal biopsy specimens from the final 50 cases. The cohort was allocated into the H3Cit-positive (any H3Cit-positive cells present in renal tissue) and H3Cit-negative (no positive cells) groups (Fig. 1). In 42 of the 50 cases (84%), H3Cit-positive cells were observed in the glomeruli and/or interstitium. Table 1 summarizes the clinical characteristics of all 50 patients, including those who had initiated therapy for AAV prior to renal biopsy and those who were treatment-naive at biopsy. The median age of the entire cohort was 73 years, consistent with a predominantly older population. The median sCr was 1.3 mg/dl; overall, the cohort exhibited renal dysfunction accompanied by proteinuria and hematuria. Comparing the H3Cit-positive and -negative groups, no significant differences were found in age, sex, or prevalence of hypertension. Additionally, the proportions of patients who had initiated AAV therapy before biopsy versus those who were treatment-naïve did not differ significantly between the two groups. Renal function tended to be worse in the H3Cit-positive group, reflected by higher sCr and lower eGFR; however, these differences were not statistically significant. However, uβ_2_-MG and the NAG index, known as markers of tubulointerstitial injury, were significantly higher in the H3Cit-positive group (Table 1 and Fig. 2). MPO-ANCA titers and CRP levels were comparable between the groups; however, complement C3 and C4 tended to be lower in the H3Cit-positive group; CH50 was significantly reduced (Table 1). Next, we then limited the analysis to treatment-naïve patients at biopsy, excluding those who had already begun AAV therapy (Table 2). In this subset, the H3Cit-positive group again showed significantly higher uβ_2_-MG and NAG index than did the negative group, whereas complement activity was lower only as a nonsignificant trend. Extrarenal manifestations and induction regimens are summarized in Table 3. No significant between-group differences were found in the frequencies of ear–nose–throat, pulmonary, or neurologic involvement. Although intravenous steroid pulse therapy tended to be used more frequently in the H3Cit-positive group, the proportions receiving oral corticosteroids versus intravenous steroid pulse therapy did not differ significantly between the groups.

**Table 1:** Baseline characteristics and laboratory findings in patients with AAV according to H3Cit status.

	H3Cit all (*n* = 50)	H3Cit+ (*n* = 42)	H3Cit− (*n* = 8)	*P-*value	*q-*value
Age (years)	73.0 [64.8–76.0]	73.5 [64.0–76.0]	68.5 [65.5–79.8]	1.000	1.000
Sex (F/M), *n*	33/17	28/14	5/3	.999	1.000
Hypertension, *n*	22	18	4	–	–
Treatment initiated at biopsy (yes/no), *n*	17/33	15/27	2/6	–	–
sCr (mg/dl)	1.30 [0.74–1.72]	1.53 [1.07–2.58]	1.19 [0.64–1.98]	.250	0.438
eGFR (ml/min per 1.73 m^2^)	32 [16–60]	31.5 [14–53]	44 [26–86]	.223	0.446
Hb (mg/dl)	9.6 [8.4–10.9]	9.6 [8.6–10.6]	10.6 [7.9–13.0]	.321	0.409
Alb (mg/dl)	2.9 [2.4–3.3]	2.9 [2.3–3.2]	3.2 [2.8–3.9]	.072	0.202
CRP (mg/dl)	1.42 [0.19–8.02]	1.57 [0.20–8.59]	0.43 [0.15–4.55]	.308	0.479
MPO-ANCA (IU/ml)	169 [48–397]	161 [46–381]	179 [56–426]	.874	1.000
C3 (mg/dl)	110 [98–132]	109 [94.5–129]	123 [105–138]	.119	0.278
C4 (mg/dl)	30 [23–37]	28 [22–33]	35 [29–39]	.072	0.252
CH50 (U/ml)	51 [43–59]	47 [38–58]	59 [52–60]	**.009**	0.063
uβ₂-MG (mg/dl)	4617 [276–33 427]	17 318 [724–34 961]	157 [118–818]	**.002**	**0.028**
NAG index (U/g Cr)	26.1 [19.1–65.4]	25.5 [14.6–36.5]	9.9 [7.4–17.9]	.**010**	**0.047**
U-pro (g/g Cr)	0.86 [0.51–1.79]	0.94 [0.54–1.79]	0.65 [0.19–3.77]	.315	0.441
U-RBC (/HPF), *n*					
<0 to 5	7	7	0	–	–
<50	26	20	6	–	–
≧50	10	9	1	–	–
≧100	7	6	1	–	–

Values are presented as median (25%–75% quartile) or number of patients, as appropriate. *P*-values were calculated using the Mann–Whitney U test for continuous variables and Fisher’s exact test for categorical variables. *Q*-values represent *P*-values adjusted for multiple comparisons using the false discovery rate method.

F, female; M, male; PSL, prednisolone; IVCY, intravenous cyclophosphamide; RTX, rituximab; sCr, serum creatinine; eGFR, estimated glomerular filtration rate; Hb, hemoglobin; Alb, albumin; CRP, C-reactive protein; MPO-ANCA, myeloperoxidase antineutrophil cytoplasmic antibody; uβ_2_-MG, urinary β_2_-microglobulin; NAG index, urinary N-acetyl-β-D-glucosaminidase index; U-pro, urinary protein; U-RBC, urinary red blood cell; HPF, high-power field.

**Table 2:** Baseline characteristics and laboratory findings in untreated patients according to H3Cit status.

	H3Cit+ (*n* = 27)	H3Cit− (*n* = 6)	*P-*value
Age (years)	74 [56–77]	69 [66–77]	.963
Sex (F/M), *n*	18/9	4/2	1.000
sCr (mg/dl)	1.29 [0.71–1.87]	0.75 [0.60–1.55]	.145
eGFR (ml/min per 1.73 m^2^)	34 [25–69]	61 [34–92]	.102
Hb (mg/dl)	9.7 [8.8–10.6]	11.8 [9.7–13.4]	.065
Alb (mg/dl)	3.0 [2.2–3.5]	3.5 [3.0–4.2]	.072
CRP (mg/dl)	5.66 [0.26–10.06]	0.43 [0.18–5.48]	.234
MPO-ANCA (IU/ml)	213 [49–640]	179 [70–401]	.815
C3 (mg/dl)	103 [91–134]	123 [102–146]	.153
C4 (mg/dl)	30 [23–36]	35 [26–40]	.293
CH50 (U/ml)	47.7 [42–57.9]	59.1 [51.5–59.7]	.072
uβ₂-MG (mg/dl)	1773 [287–27 900]	119.5 [119.5–1007.3]	**.031**
NAG index (U/g Cr)	26.1 [13.7–65.4]	9.3 [6.7–14.1]	**.017**
U-pro (g/g Cr)	1.15 [0.64–1.75]	0.37 [0.14–1.77]	.084
U-RBC (/HPF), *n*			
<0 to 5	2	0	–
<50	13	4	–
≧50	7	1	–
≧100	5	1	–

Values are presented as median (25%–75% quartile) or number of patients, as appropriate. *P*-values were calculated using the Mann–Whitney U test for continuous variables and Fisher’s exact test for categorical variables.

F, female; M, male; PSL, prednisolone; IVCY, intravenous cyclophosphamide; RTX, rituximab; sCr, serum creatinine; eGFR, estimated glomerular filtration rate; Hb, hemoglobin; Alb, albumin; CRP, C-reactive protein; MPO-ANCA, myeloperoxidase antineutrophil cytoplasmic antibody; uβ_2_-MG, urinary β_2_-microglobulin; NAG index, urinary N-acetyl-β-D-glucosaminidase index; U-pro, urinary protein; U-RBC, urinary red blood cell; HPF, high-power field.

**Table 3:** Distribution of extrarenal manifestations and induction therapy.

	H3Cit+ (*n* = 42)	H3Cit− (*n* = 8)	*P-*value
Manifestations, *n*			
Pulmonary involvement	12	2	.687
Ear, nose, and throat	3	1	.670
Neurologic involvement	3	1	.413
Induction therapy			
Oral steroid, *n*	12	3	.683
Oral steroid + IVCY, *n*	4	2	.242
Oral steroid + RTX, *n*	0	1	.160
IV steroid-pulse, *n*	7	1	1.000
IV steroid pulse + IVCY, *n*	9	1	1.000
IV steroid pulse + Rituximab, *n*	9	0	.322
No treatment	1	0	1.000

Values are presented as the number of patients. *P*-values were calculated using Fisher’s exact test for categorical variables. No statistically significant differences were observed between the H3Cit-positive and H3Cit-negative groups for any parameter.

H3Cit, citrullinated histone H3; IVCY, intravenous cyclophosphamide; RTX, rituximab; IV, intravenous.

### Pathological characteristics

**Figure 3:**
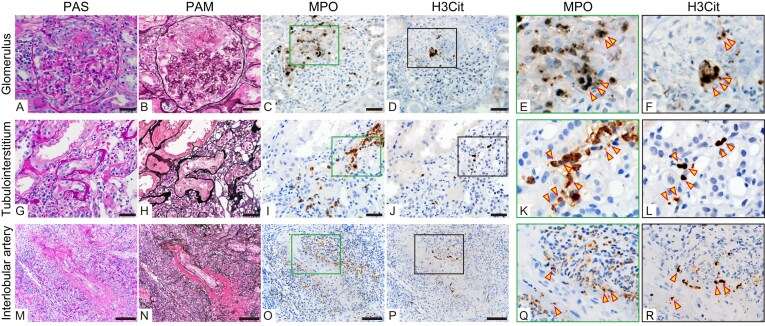
Immunohistochemical detection of H3Cit- and MPO-positive cells in renal biopsy specimens from patients with AAV. (A–F) Glomerular necrotizing lesions with crescent formation: PAS (A) and PAM (B) staining show a glomerulus with fibrinoid necrosis of the tuft and cellular crescents. Serial sections of the same glomerulus stained for MPO (C) and H3Cit (D) demonstrate numerous MPO-positive cells within the necrotic and crescentic areas, among which several are also H3Cit-positive, indicating NET formation in active glomerular lesions. Panels A–D represent serial sections of the same glomerulus. Panels E and F show higher magnification images of the boxed areas in panels C and D, respectively. Scale bar, 50 µm. (G–L) Tubulointerstitial inflammation with PTC-itis: PAS staining (G) and PAM staining (H) reveal inflammatory cell infiltration within peritubular capillaries and extension of inflammation into the surrounding tubular epithelial cells and interstitium. Panels G–J represent serial sections of the same area. MPO immunostaining (I) shows numerous MPO-positive cells in these inflamed peritubular capillaries and interstitium. H3Cit immunostaining (J) demonstrates that a subset of these MPO-positive cells is H3Cit-positive, consistent with NET formation in the tubulointerstitial compartment. Panels K and L show higher magnification images of the boxed areas in panels I and J, respectively. Scale bar, 50 µm. (M–R) Necrotizing arteritis: PAS staining (M) and PAM staining (N) show an interlobular artery with marked inflammatory cell infiltration, disruption of the vessel wall, and features of severe necrotizing arteritis. Serial sections from the same lesion stained for MPO (O) reveal numerous MPO-positive cells within the inflamed arterial wall. H3Cit immunostaining (P) demonstrates that some of these MPO-positive cells are H3Cit-positive, localized to areas of fibrinoid necrosis. Panels Q and R show higher magnification images of the boxed areas in panels O and P, respectively. Scale bar, 100 µm. Arrowheads indicate H3Cit-positive cells identified in corresponding serial sections.

Figure 3 illustrates the IHC results for MPO and H3Cit on serial sections of paraffin-embedded renal biopsy specimens, performed to clarify the relationship between H3Cit-positive cells and histological activity.

In all 42 H3Cit-positive cases, positive cells were detected in the interstitium; in 23 of these, they were also observed within the glomeruli. In glomerular lesions, MPO-positive cells, including a subset that was H3Cit-positive, were identified predominantly in necrotic lesions and crescent formation area with marked leukocyte infiltration (Fig. 3). Of the 23 cases of glomerular H3Cit-positive cells, necrotizing lesions were observed in 13; in 8 cases of these, H3Cit-positive cells were identified specifically within the necrotic areas. The H3Cit-positive group showed a significantly higher frequency of crescent formation compared with the negative group (Table 4). However, no correlation was found between the number of glomerular H3Cit-positive cells and rate of crescent formation (data not shown). There was also no difference between the two groups in the proportion of globally sclerotic glomeruli.

**Table 4:** Comparison of renal histopathological features between H3Cit-positive and H3Cit-negative patients.

	H3Cit+ (*n* = 42)	H3Cit− (*n* = 8)	*P-*value
Presence of glomerular necrosis, *n*	16	1	.767
Glomerular crescent formation rates (%)	11.1 [3.4–21.7]	0	**.001**
Global sclerotic glomeruli rates (%)	20.1 [5.9–32.2]	39.2 [1.7–68.6]	.367
Interstitial fibrosis rates (%)	35.0 [20.0–60.0]	25 [11.3–40.0]	.229
Presence of PTC-itis, *n*	30	1	**.002**
Presence of tubulitis, *n*	24	1	.253
Presence of arteritis, *n*	7	0	.914
Atherosclerosis score	1.5 [1.0–2.3]	1.5 [1.0–2.8]	.719
C3 positivity (IF), *n*	12	4	.833

Values are presented as median (25%–75% quartile) or number of patients, as appropriate. *P*-values were calculated using the Mann–Whitney U test for continuous variables and Fisher’s exact test for categorical variables.

H3Cit, citrullinated histone H3; IF, immunofluorescence; PTC-itis, peritubular capillaritis.

**Figure 4:**
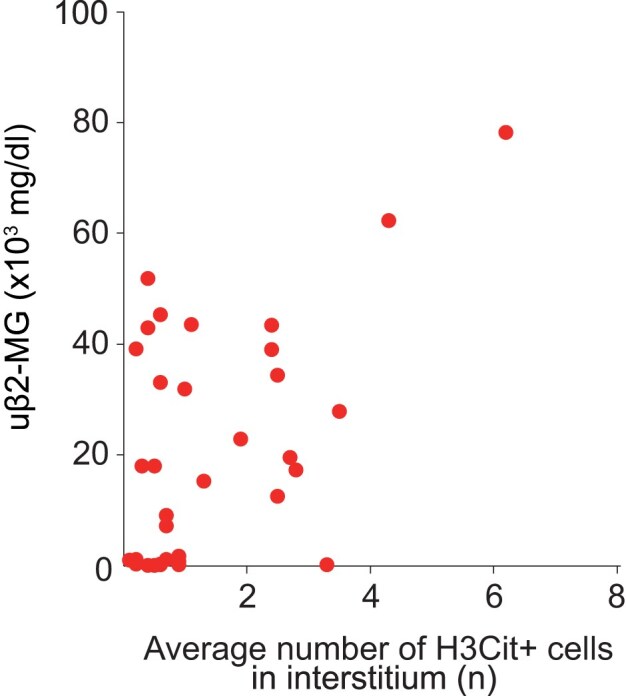
Correlation between interstitial H3Cit-positive cells counts and uβ_2_-MG levels in patients with AAV. The scatter plot shows a positive correlation between the average number of interstitial H3Cit-positive cells per 10 randomly selected HPFs and uβ₂-MG levels (mg/dl) (Spearman’s rank correlation coefficient, *R* = 0.499, *P* = .048).

Focusing on tubulointerstitium, inflammatory cell infiltration was noted in the PTC and interstitium (Fig. 3), with frequent detection of MPO-positive cells, a subset of which were H3Cit-positive. The H3Cit-positive group showed a significantly higher frequency of cases of PTC-itis and a trend toward a higher frequency of tubulitis (Table 4). Based on the clinical data presented in Table 2 and Fig. 2, which show significantly higher uβ_2_-MG and NAG index values, markers of tubular and interstitial injury, in the H3Cit-positive group, we further examined their association with the number of H3Cit-positive cells. A positive correlation was found between the number of interstitial H3Cit-positive cells and uβ_2_-MG levels (Fig. 4). However, no correlation was found between the number of interstitial H3Cit-positive cells and NAG index value (data not shown). Furthermore, arteritis was confirmed in seven cases; notably, H3Cit-positive cells were consistently present in all arteritic lesions (Fig. 3 and Table 4). No between-group differences were found in arteriosclerosis scores or extent of interstitial fibrosis. Similarly, the proportion of cases showing glomerular C3 positivity on routine diagnostic IF study did not differ between the groups.

## DISCUSSION

We investigated whether IHC for H3Cit, a marker of NETs, in renal biopsy specimens from patients with AAV could serve as a useful indicator of disease activity. A key novelty of this study lies in its comprehensive analysis of the relationship between H3Cit staining and both clinical parameters and pathological findings. In our previous work, we visualized H3Cit-positive cells in FFPE sections of renal biopsy specimens and compared various types of nephritis accompanied by neutrophil infiltration. We demonstrated that the frequency of H3Cit-positive cells was significantly higher in patients with AAV than in those with other conditions, with interstitial H3Cit-positive cells being particularly characteristic of AAV. Based on these findings, the present study focused exclusively on AAV, expanded the sample size, and found that H3Cit-positive cells were present in renal tissue in more than 80% of cases, which is a remarkably high proportion.

In patients with AAV, several clinical studies have demonstrated that more severe renal impairment at diagnosis is associated with a higher risk of end-stage kidney disease and mortality [24–26]. The severity and prognosis of renal involvement in AAV are often assessed on the basis of histopathological findings; various prognostic scoring systems have been proposed. The prognostic value of quantifying pathological features was first reported in the 1990s [27, 28]. Additionally, subsequent studies have confirmed renal biopsy findings as reliable predictors of renal outcome [29]. Given these prognostic implications, highlighting that characteristic active glomerular lesions in patients with AAV include glomerular basement membrane disruption, necrotizing changes, and cellular or fibrocellular crescent formation is important. In the tubulointerstitium, tubulitis and PTC-itis are also recognized as indicators of active lesions in patients with AAV [30]. These pathological features were also evaluated in the present study to explore their association with H3Cit-positive cells.

In recent years, the ANCA Renal Risk Score, which incorporates the percentage of normal glomeruli, degree of IFTA, and eGFR, has been validated in prospective multicenter studies, enabling a more objective assessment of renal prognosis [31, 32]. In the present study, comparison of renal function at the time of biopsy revealed a trend toward higher sCr levels and lower eGFR values in the H3Cit-positive group; however, these differences did not reach statistical significance between the two groups. However, uβ_2_-MG and the NAG index were significantly higher in the H3Cit-positive group; this trend remained unchanged when the analysis was limited to untreated cases. uβ_2_-MG and NAG are established markers for assessing tubular and interstitial injury and also useful for the early diagnosis of acute kidney injury, being considered more sensitive compared with sCr [33–35]. In this study, the significantly higher levels of these biomarkers in the H3Cit-positive group might indicate a greater degree of tubular and interstitial injury, compared with the negative group, and could also reflect the presence of acute lesions. Pathological comparison further revealed a significantly higher frequency of PTC-itis in the H3Cit-positive group, whereas no association was found with chronic lesions such as interstitial fibrosis. Moreover, a positive correlation was observed between the number of interstitial H3Cit-positive cells and uβ_2_-MG levels, suggesting that the presence of and particularly the increased number of H3Cit-positive cells infiltrating the interstitium may be associated with the extent of interstitial injury. These observations suggest that NET formation in inflamed peritubular capillaries might contribute to tubular–interstitial injury in patients with AAV, potentially through extending vascular inflammation into the surrounding interstitium. Regarding glomerular lesions, the H3Cit-positive group also showed a significantly higher crescent formation rate, wherein H3Cit-positive cells were frequently observed within necrotic lesions. Notably, H3Cit-positive cells were present in all sites of severe arteritis accompanied by necrosis. In contrast, no association was observed with globally sclerotic glomeruli, which may simply reflect the lack of inflammatory cell infiltration in such chronic lesions. Collectively, these observations indicate that H3Cit-positive cells are closely associated with active vasculitic lesions.

NET formation has also been implicated in other autoimmune diseases such as systemic lupus erythematosus. Building upon our previous comparative findings, H3Cit-positive cells were also observed in lupus nephritis. However, they were less frequent and showed a different distribution pattern. In the present study, we further extend these observations by demonstrating that the number of H3Cit-positive cells is associated with clinical and histopathological indicators of disease activity in AAV. These findings suggest that H3Cit-positive cells may represent not only a disease-associated feature but also a marker reflecting the activity of vasculitic lesions.

In the present study, MPO immunostaining was used to identify inflammatory cells, as MPO is commonly employed as a marker of neutrophils in tissue sections. However, MPO expression is not entirely restricted to neutrophils and has also been reported in certain macrophage populations under inflammatory conditions [17, 36]. Moreover, extracellular trap formation is not limited to neutrophils, as macrophages can release METs. Accordingly, extracellular trap formation observed in AAV lesions may not be exclusively neutrophil-derived. In this study, H3Cit immunostaining provided a practical approach to visualize extracellular trap formation in paraffin-embedded renal biopsy specimens and to explore its relationship with disease activity. Further studies incorporating more precise cell-type identification may clarify the specific cellular contributors to extracellular trap formation in AAV.

NETs have long been reported to interact with the complement system, thereby amplifying inflammation [37]. Regarding the association between AAV and complements, previous studies have shown that lower serum C3 levels are associated with poorer renal outcomes, and that serum C3 levels negatively correlate with the proportion of glomeruli exhibiting crescent formation [38–41]. Elevated levels of serum complement activation products, such as C3a, C5a, and soluble C5b-9, have also been reported to correlate with disease activity [42, 43]. Notably, the complement anaphylatoxin C5a has been shown to further prime ANCA-activated neutrophils, thereby amplifying inflammation, suggesting that crosstalk between complement activation and NET formation may play a central role in the pathogenesis of the disease [43, 44]. In the present study, the H3Cit-positive group tended to show lower complement levels, suggesting a possible association between complement and disease activities. At the tissue level, we compared the frequency of cases showing glomerular C3 deposition on routine diagnostic IF between the two groups; however, no significant difference was found. As complement deposition and H3Cit staining were not evaluated in the same glomeruli, a direct relationship between glomerular complement deposition and H3Cit-positive cells could not be assessed at the individual glomerulus level.

## LIMITATIONS

This study has some limitations. First, the relatively small sample size and the retrospective single-center design might have limited the statistical power and carry an inherent risk of selection and information bias. Relatedly, heterogeneity in disease stage and treatment history among the patients might also have influenced the results. Future studies using human renal tissue samples from multiple centers are warranted to elucidate in greater details the local interactions between NETs and the complement system.

Next, several limitations are related to the histopathological assessment. The assessment of histological lesions and complement deposition was semiquantitative, which might have introduced variability related to interobserver differences and staining conditions. In addition, single immunostaining on paraffin-embedded renal biopsy sections enabled evaluation of the overall distribution of H3Cit-positive cells and their relationship with active vasculitic lesions, but precise cell-type identification using double or triple immunostaining was not feasible. Therefore, MPO-positive and H3Cit-positive cells could not be definitively distinguished as neutrophils or macrophages.

## CONCLUSION

The presence of H3Cit-positive cells in renal tissue was associated with disease activity in patients with AAV, including active glomerular and tubulointerstitial lesions. These findings support the potential utility of H3Cit immunostaining as a pathological marker of disease activity in patients with AAV and as an adjunctive tool for evaluating disease activity at the time of renal biopsy. Future prospective studies are warranted to validate the clinical utility of H3Cit staining as a biomarker for treatment response and long-term renal outcome. Moreover, H3Cit immunostaining might aid in assessing treatment responsiveness and prognosis, contribute to elucidating the pathogenic role of NETs in patients with AAV, and inform the development of therapeutic strategies targeting NET formation pathways.

## Supplementary Material

sfag110_Supplemental_Files

## Data Availability

The data that support the findings of this study are available from the corresponding author upon reasonable request.
