# Supplementary material for: Citrullinated histone H3 identifies neutrophil extracellular trap formation and correlates with renal disease activity in ANCA-associated vasculitis
Source: Clin Kidney J. 2026 Apr 6;19(5):sfag110. doi: 10.1093/ckj/sfag110 (PMC13134445; doi:10.1093/ckj/sfag110)
Supplement: sfag110_Supplemental_Files [file sfag110_supplemental_files.zip › ANCA_paper_Legend of Supplemental Figure_20260227.docx]

**Legend of Supplemental Figure**

**Supplemental Figure 1. Validation of H3Cit immunostaining using two independent antibodies.**

Panels A–C show immunohistochemical staining using an anti-H3Cit antibody (ab281584; Abcam, Cambridge, UK), whereas panels D–F show staining using an independent anti-H3Cit antibody (#97272; Cell Signaling Technology, Danvers, MA, USA). Panels A and D, B and E, and C and F represent serial sections from the same tissue regions. H3Cit-positive cells (arrowheads in A and D) and H3Cit-positive cells within glomeruli outlined by red dotted circles (C and F) were consistently detected in corresponding regions with both antibodies, demonstrating comparable staining patterns.

Scale bar, 50 μm.
