# Supplementary figures and images for: Citrullinated histone H3 identifies neutrophil extracellular trap formation and correlates with renal disease activity in ANCA-associated vasculitis
Source: Clin Kidney J. 2026 Apr 6;19(5):sfag110. doi: 10.1093/ckj/sfag110 (PMC13134445; doi:10.1093/ckj/sfag110)

Supplemental Figure 1

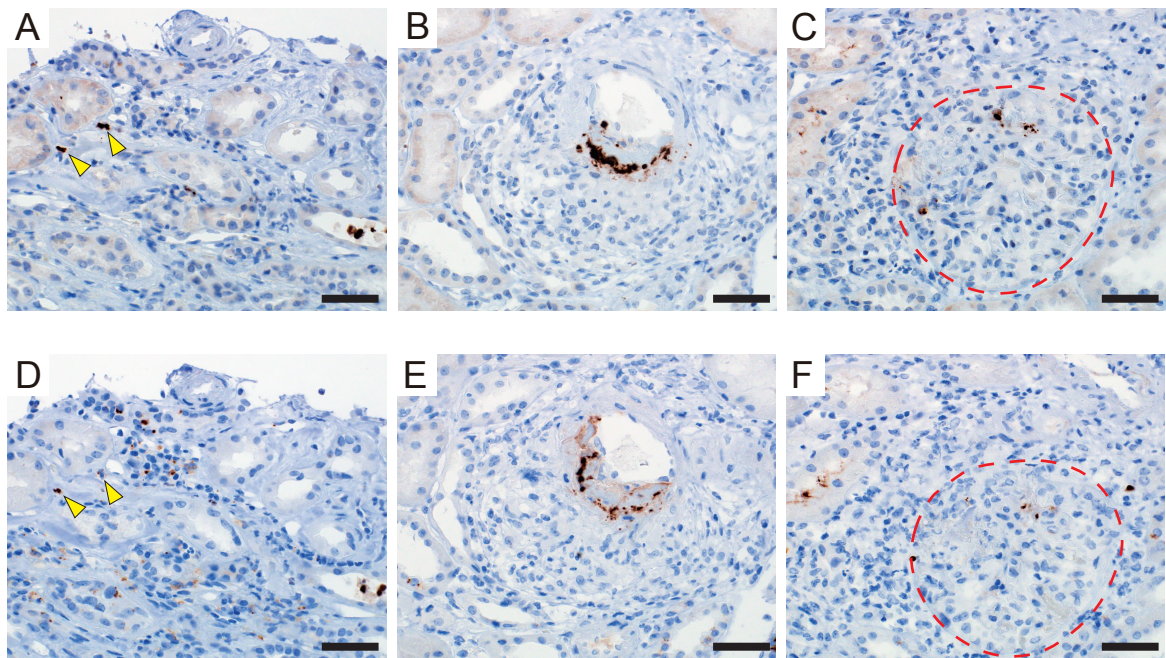

Supplement: sfag110_Supplemental_Files [file sfag110_supplemental_files.zip › Supplemental Figure 1 20260226.pdf]
